# Regulatory B cell Il-10 production is diminished in juvenile dermatomyositis

**DOI:** 10.1186/1546-0096-12-S1-P86

**Published:** 2014-09-17

**Authors:** Christopher Piper, David Bending, Hemlata Varsani, Katie Arnold, Lucy Wedderburn, Claudia Mauri, Kiran Nistala

**Affiliations:** 1 Centre for Rheumatology, Division of Medicine, University College London, London, UK; 2 Infection, Inflammation and Rheumatology Section UCL Institute of Child Health, University College London, London, UK

## Introduction

Juvenile dermatomyositis (JDM) is a childhood autoimmune disease characterised by proximal muscle weakness and cutaneous manifestations. Previous studies have identified an increase in circulating B cells in JDM patients, but their provenance and functional characteristics have not been examined. In this study we investigated whether an immature B cell subset (CD24^hi^CD38^hi^), known to be enriched for interleukin (IL)-10 producing regulatory B cells (Breg)**^1^**, accounted for the expansion of circulating B cells seen in JDM. The aryl hydrocarbon receptor (AhR) is a ligand based transcription factor, which induces IL-10 expression in T cells**^2^**. We investigated the effects of modulating the AhR pathway on IL-10 expression in B cells.

## Objectives

- To characterise the peripheral blood B cell compartment in JDM patients with active disease and in disease remission (according to the PRINTO criteria).

- To test the capacity of B cells from JDM patients to produce IL-10 and ask if modulation of the AhR pathway alters B cell IL-10.

## Methods

54 patients were recruited through the UK JDM Cohort and Biomarker Study. B cell subpopulations from peripheral blood mononuclear cells (PBMC) isolated from healthy controls (HC) and JDM patients were analysed by flow cytometry using the surface markers CD19, CD24, CD38 and CD27. To detect B cell IL-10, PBMC were stimulated for 72 hours with CD40 ligand (CD40L) transfected CHO cells or the toll like receptor 9 agonist CpG (ODN 2006) +/- Ahr antagonist (CH-223191), together with PMA and ionomycin for the last 5 hours in the presence of Brefeldin A. Cells were then stained for CD19 and intra-cellular IL-10, which was detected by flow cytometry.

## Results

PBMC from JDM patients with active disease had a significantly lower frequency of CD24hiCD38hi Breg when compared to inactive JDM patients (median of 7.6% vs 12.9% respectively, p=0.0109). Active patients had a lower frequency of IL-10 producing B cells compared to inactive patients and child controls (mean of 12.1%, 15.8% and 18.6% respectively), but this was only observed following stimulation with CD40L and not CpG. Inhibition of AhR following CD40L stimulation augmented IL-10 production in JDM B cells, restoring it to normal levels. Blocking AhR had no effects on CpG induced B cell IL-10.

## Conclusion

These data identify a reduction in Breg in JDM patients with active disease that was associated with defective CD40L induced IL-10, when compared to child controls. This defect was reversed following blockade of AhR. These results suggest that over-activity of the AhR pathway may contribute to the pathophysiology of JDM, by attenuating Breg function.

## Disclosure of interest

None declared.
